# Evolution of long centromeres in fire ants

**DOI:** 10.1186/s12862-016-0760-7

**Published:** 2016-09-15

**Authors:** Yu-Ching Huang, Chih-Chi Lee, Chia-Yi Kao, Ni-Chen Chang, Chung-Chi Lin, DeWayne Shoemaker, John Wang

**Affiliations:** 1 Biodiversity Research Center, Academia Sinica, Taipei, Taiwan; 2 Laboratory of Insect Social Evolution, The Rockefeller University, New York, NY USA; 3 Department of Biology, National Changhua University of Education, Changhua, Taiwan; 4 United States Department of Agriculture, Agricultural Research Service, Gainesville, FL USA; 5 Entomology and Plant Pathology Department, University of Tennessee, Knoxville, TN USA

**Keywords:** Centromere, Centromere drive, *Solenopsis invicta*, *Solenopsis geminata*, Fire ant

## Abstract

**Background:**

Centromeres are essential for accurate chromosome segregation, yet sequence conservation is low even among closely related species. Centromere drive predicts rapid turnover because some centromeric sequences may compete better than others during female meiosis. In addition to sequence composition, longer centromeres may have a transmission advantage.

**Results:**

We report the first observations of extremely long centromeres, covering on average 34 % of the chromosomes, in the red imported fire ant *Solenopsis invicta*. By comparison, cytological examination of *Solenopsis geminata* revealed typical small centromeric constrictions. Bioinformatics and molecular analyses identified *CenSol*, the major centromeric satellite DNA repeat. We found that *CenSol* sequences are very similar between the two species but the *CenSol* copy number in *S. invicta* is much greater than that in *S. geminata*. In addition, centromere expansion in *S. invicta* is not correlated with the duplication of *CenH3*. Comparative analyses revealed that several closely related fire ant species also possess long centromeres.

**Conclusions:**

Our results are consistent with a model of simple runaway centromere expansion due to centromere drive. We suggest expanded centromeres may be more prevalent in hymenopteran insects, which use haplodiploid sex determination, than previously considered.

**Electronic supplementary material:**

The online version of this article (doi:10.1186/s12862-016-0760-7) contains supplementary material, which is available to authorized users.

## Background

Centromeres serve as the fundamental chromosome structure responsible for accurate chromosome segregation during eukaryotic cell division. Most eukaryotic chromosomes are monocentric, having microtubule attachment domains restricted to a small constriction zone. In contrast, holocentric chromosomes have microtubule binding domains along the entire length of the chromosome and have independently evolved many times [1–3].

Monocentric and holocentric chromosomes have been extensively studied, but less attention has been given to centromeres with intermediate structures. Centromeres with longer constrictions occur after Robertsonian fusions [4] as well as in atypical situations such as in hybrids and cancer cell lines [5, 6]. There are also cases where centromeres appear to have undergone extreme expansion. For example, chromosomes with longer “compound centromeres” have been reported in a few mammalian species, including the muntjac [7] and mouse [8]. Similarly extremely large primary constrictions, or “metapolycentric” centromeres, have been observed in legume plants [9, 10].

The evolution of such large centromeres may represent cases of “centromere drive” [11, 12]. Female meiosis in plants and animals is asymmetric with only one of the four meiotic products entering the egg. Under the centromere drive model, centromeric alleles on chromosomes, especially longer alleles, attach more strongly or efficiently to spindle microtubules at the kinetochore compared with other alleles and thereby gain a transmission advantage into the egg. This model also may explain the observed rapid evolution of centromeric DNA sequences among lineages, which are generally highly repetitive satellites, because such sequences that bind stronger to the spindle would be similarly preferentially transmitted.

Success of chromosomes as a result of centromere drive in female meiosis simultaneously can have a negative influence on male meiosis. In contrast to female meiosis, all four meiotic products become gametes in males. Unequal binding at the kinetochore during chromosome segregation may trigger cell cycle checkpoints that are deleterious, such as reduced fertility or aneuploidy [13–15]. As a consequence of these deleterious effects, there should be selection for compensatory mutations (suppressors) that restore meiotic parity [12]. Consistent with this idea, kinetochore proteins, including the CenH3 protein, which is the histone H3 variant that binds and defines centromeres, also evolve rapidly, presumably to compensate for or suppress chromosome segregation defects [11, 12]. In addition to faster sequence evolution, duplication of *CenH3* has occurred in some legume species, but only in those with expanded centromeres [9]. Once suppression has evolved, continued competition for the oocyte during female meiosis may select for different but “stronger” primary centromeric sequences, leading to repeated cycles of expansions and collapses [12, 16]. Additionally, deleterious mutations that become linked to driving centromeres likely counterbalance unlimited expansion [14, 16, 17].

The recent discovery of metapolycentric chromosomes reveals that there is likely a continuum in centromere structures between monocentric and holocentric chromosomes [9, 10]. We first noticed unusual chromosome structures in the red imported fire ant *Solenopsis invicta* in a FISH experiment [18] and decided to explore further. In this article, we report the first observations of extremely long centromeres in *S. invicta*. We conducted cytological, bioinformatics, molecular, and comparative analyses to identify and characterize *CenSol*, the major centromeric satellite DNA repeat in fire ants. Our results are consistent with a model of simple runaway centromere expansion due to centromere drive for the evolution of long centromeres in fire ants.

## Results

### Centromeres are larger in *S. invicta* than *S. geminata*

**Fig. 1:**
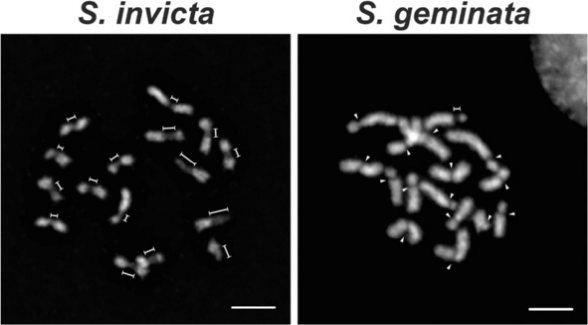
Contrasting centromere morphologies in *S. invicta* and *S. geminata*. The centromere structure was revealed by DAPI staining of metaphase chromosomes. The elongated (dimension lines) and the small (arrowheads) primary constrictions are indicated. Scale bars, 5 μm

We used DAPI to stain metaphase chromosomes and found that, in contrast to typical monocentric chromosomes with a narrow constriction at the centromere, every *S. invicta* chromosome exhibited long primary constrictions (Fig. 1). Primary constrictions spanned an average of 34 % of the chromosome length (constrictions among chromosomes range from 17.3 ± 1.3 to 54.8 ± 6.1 %). We used a quantification method [9] that adjusts for the lower DNA intensity and narrowness at the constricted region to estimate the proportion of the chromosome represented by the centromere. With the qualification that condensation patterns may be affected by the specific chromosomal preparation method used, we estimated that the primary constrictions accounted for ~3.6 Mb of individual chromosomes (1.8 ± 0.5 to 6.3 ± 1.3 Mb), and in total covered ~58 Mb (12 %) of the predicted haploid genome size of 484 Mb [19].

We examined centromere structure in the closely related tropical fire ant *S. geminata* to test whether this extended primary constriction was specific to *S. invicta.* Surprisingly, 15 of the 16 *S. geminata* chromosomes showed the typical monocentric morphology with small primary constrictions; a single exception exhibited an elongated constriction (Fig. 1). The primary constrictions on the 15 typical chromosomes spanned an average of only 11 % of the individual chromosome length (6.5 ± 1.3 % to 16.0 ± 1.8 %), which was about three-fold less than that of *S. invicta*. The centromere on the exceptional chromosome spanned 23.8 % (± 5.9 %). The centromere morphology found on all *S. invicta* chromosomes and a single chromosome of *S. geminata* is similar to that described as ‘compound centromeres’ or ‘metapolycentric’ chromosomes [7–10].

### Candidate centromeric satellite sequences in fire ants

The most prevalent tandem repeat, or satellite, in a genome generally is assumed to be the candidate centromeric repeat [20, 21]. We followed an established bioinformatics pipeline [20] to identify high copy tandem satellites from the draft genomes of *S. invicta* and *S. geminata* (Additional files 1 and 2). The top ten satellites and their summary statistics for both species are shown in Additional file 3: Table S1. We compared the sequences by BLAST similarity searches to identify shared satellites within the two top-ten lists. We found nine repeats were shared between the ant genomes, with only the top two having identical ranks (Additional file 3: Table S1).

Centromeres are composed of only one dominant repeat in many species, but some species do have more than one type of repeat [22, 23]. Thus, we focused on the top two satellites for both *S. invicta* and *S. geminata*. The top satellite for both fire ants was a similar 109 bp repeat. These monomers had minor sequence and length variation, which is typical of centromeric satellites in other species [24–26]. The average GC content was 39.4 and 38.5 % in *S. invicta* and *S. geminata*, respectively, which is compatible with observations suggesting a slight preference for AT-rich centromeric satellites in animals [20]. This repeat showed no significant similarity to any dominant tandem repeat from 282 species, including the candidate centromeric repeats in four ants [20], and also no similarity to known transposons or satellite sequences (BLASTN against NCBI nr best hit: bit score = 41.0, E-value = 3.9; Repbase databases: no hits).

The second most abundant satellite was 139 bp in *S. invicta* and 138 bp in *S. geminata*. Although the modal lengths differed by 1 bp, the repeats in both clusters had an average identity of 89.7 %. Similarly, the counts of the second ranked satellites had a dramatic drop (~13-fold in *S. invicta*, ~4-fold in *S. geminata*) compared with that of the top repeat for both genomes. We next examined the chromosomal localization of these satellites using fluorescence *in situ* hybridization (FISH) analysis to determine if they were centromeric.

### Chromosomal localization of the satellites in *S. invicta* and *S. geminata*

**Fig. 2:**
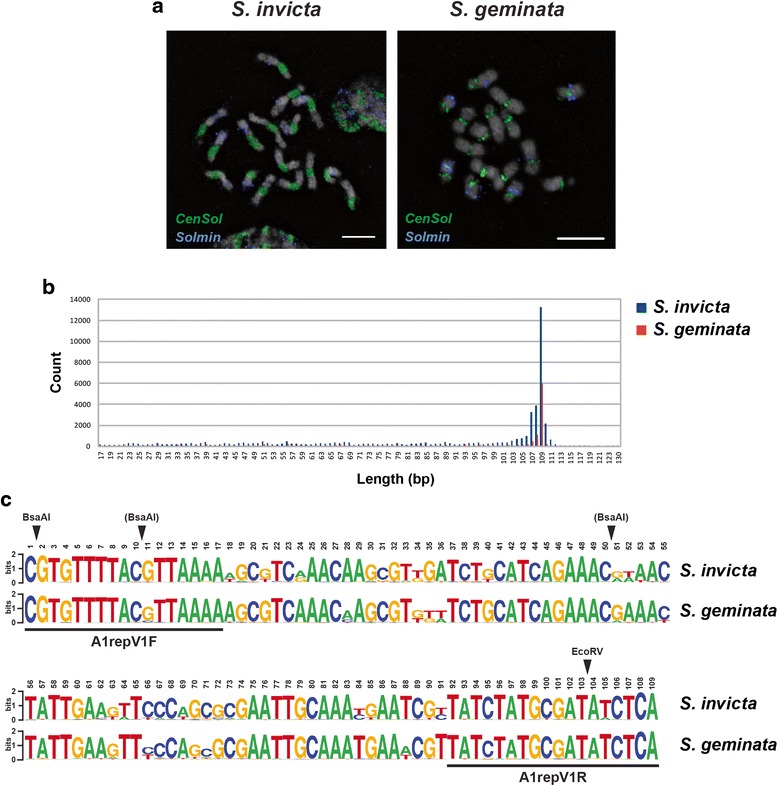
**a** Localization of the first and the second most abundant satellites on the metaphase chromosomes of *S. invicta* and *S. geminata*. FISH analysis on haploid cells combining the *CenSol* probe (green) with the second most abundant satellite (*Solmin*, blue); chromosomes counterstained with DAPI (gray). **b** Summary of the length distributions of the *CenSol* repeat in the *S. invicta* and *S. geminata* draft genomes. BLASTN hits of the consensus *CenSol* sequence against the *S. invicta* and *S. geminata* genomes were binned by sequence length. The 109 bp unit was the dominant repeat length for both ants. **c** Sequence logos generated with 10,469 and 5423 unique 109 bp *CenSol* sequences from *S. invicta* and *S. geminata*, respectively. The height of each letter is proportional to the frequency of four nucleotides, adenine (A), thymine (T), guanine (G), and cytosine (C). The total height of each stack, measured in bits, is related to the binding energy [57]. EcoRV and BsaAI restriction sites are indicated with arrowheads. The BsaAI in parenthesis indicates a less common polymorphic cutting site in the *CenSol* monomers. The locations of the A1repV1 primers are shown

**Fig. 3:**
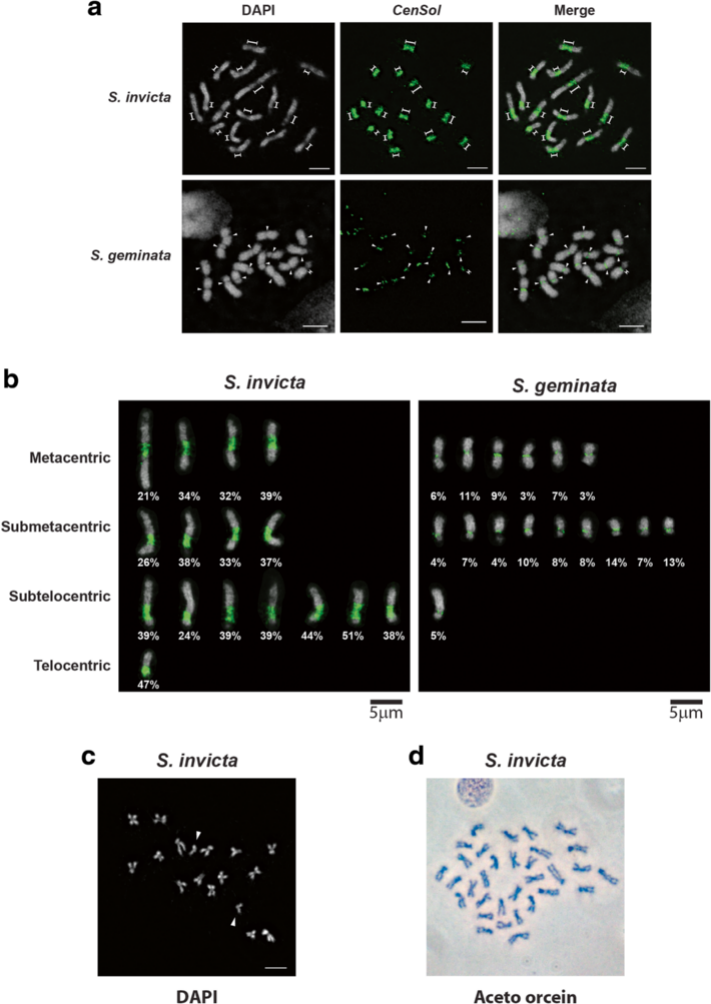
*CenSol* satellite fully localizes to the primary constriction of the haploid chromosomes in *S. invicta* and *S. geminata*. **a** and **b** FISH analysis with the *CenSol* probe (green); chromosomes counterstained with DAPI (gray). **a** The elongated (dimension lines) and the small (arrowheads) primary constrictions of chromosomes in *S. invicta* (upper panels) and *S. geminata* (lower panels) are indicated. **b** Positions of centromeres and *CenSol* coverage on chromosomes. Centromeric positions were categorized based on *CenSol* signal locations in FISH analysis. Chromosomes from *S. invicta* (left panel) and *S. geminata* (right panel) in (**a**) are artificially aligned and sorted according to the centromeric positions. The *CenSol* coverage rates (%) on each chromosome are labeled. **c** DAPI staining of the *S. invicta* chromosomes from a cell transitioning from metaphase to anaphase. Fifteen chromosome pairs are together while one pair has separated (arrowheads). **d** Aceto-orcein staining of metaphase chromosomes from male testes imaginal discs. Chromosome number is 32 likely because chromosomes come from two adjacent cells, although we cannot exclude potential diploidization which occurs in some cells of haploid male ants [58]. Scale bars, 5 μm

We performed FISH experiments on metaphase chromosomes to determine the localization of the predominant 109 bp satellite. Using a labeled monomer as the probe we found that the hybridization signals (in green) localized to all chromosomes in both males (Fig. 2a) and females (Additional file 4: Figure S2A) (ant males are haploid, 1n = 16; females are diploid, 2n = 32). Hybridization signals were restricted to one large region per chromosome rather than scattered over each chromosome suggesting a largely uninterrupted organization in the genome. Hybridization signals on average occupied 31.0 % (18.0 ± 4.8 to 44.0 ± 4.8 %) of the chromosome length in *S. invicta*. BAC-FISH revealed a pattern qualitatively indistinguishable from the FISH analysis using one monomer (Additional file 4: Figure S2B). Importantly, the hybridization location coincided with the centromeric constrictions (Fig. 3a), and hereafter we refer to this satellite as *CenSol*.

The *CenSol* hybridization signals were more restricted on all *S. geminata* chromosomes compared to *S. invicta* (Figs. 2a, 3a, and Additional file 4: Figure S2A) and only occupied an average of 9.8 % (3.5 ± 0.6 to 16.8 ± 4.9 %) of the chromosome length. For 15 of 16 chromosomes, the *CenSol* signal coincided with the centromeric constrictions. Interestingly, *CenSol* was localized only at the edge of the constriction for the one *S. geminata* chromosome with an extended centromeric constriction (Figs. 2a, 3a, one pair for females in Additional file 4: Figure S2A; marked by dimension lines). The centromeric hybridization patterns of *CenSol* were confirmed in all cells examined (*S. invicta*, *N* = 47 cells, two haploid and two diploid individuals; *S. geminata*, *N* = 45 cells, one haploid and one diploid individual).

In contrast to the centromeric localization of the *CenSol* satellite, the second most abundant satellite, hereafter called *Solmin*, was patchily distributed only on 14 and nine of the *S. invicta* and *S. geminata* chromosomes, respectively (Fig. 2a). The fluorescence signals of this repeat did not overlap with those of *CenSol* or the primary constrictions, excluding it as a centromeric or pericentric repeat. Together, these data show that the *CenSol* satellite is part, or probably all, of the centromeric satellite in *S. invicta* and all but one chromosome in *S. geminata*.

Centromeric positions can be used to describe the types of fire ant chromosomes. The centromeres of *S. invicta* previously were reported to be predominantly metacentric [27], whereas the chromosomes of *S. geminata* were metacentric and acrocentric [28]. Based on the *CenSol* signals in our FISH analysis and the cytological metaphase staining, we re-categorized *S. invicta* chromosomes into four metacentric, four submetacentric, seven subtelocentric, and one telocentric (or acrocentric) chromosomes (Fig. 3b). Chromosome classification was consistent between the *CenSol* FISH localization and our own karyotyping methods (Fig. 3c and d). On the other hand, we found six metacentric, nine submetacentric, and one subtelocentric chromosomes in *S. geminata* (Fig. 3b). Differences between the previous studies [27, 28] and ours likely can be explained by our inclusion of high-resolution FISH analysis and better chromosome resolution.

### Sequence conservation of *CenSol* in *S. invicta* and *S. geminata*

We compared *CenSol* sequences of both fire ants obtained from intact genomic arrays, which preserve the native structure, rather than the sequences from whole-genome assemblies, which may be assembled inappropriately due to their repetitive nature. Previous screening of a *S. invicta* BAC library by end sequencing revealed that 12 reads from six BAC clones (see methods) were composed of tandem repeats formed by the same monomer. Analysis using Tandem Repeat Finder [29] confirmed that the satellite DNA was composed of multiple 109 bp units, which corresponded to *CenSol* found using the bioinformatics approach. Additional screening of the *S. invicta* BAC genome library by PCR to survey *CenSol* abundance in the ant genome revealed that 66.5 % (638 of 960 clones) were positive for this satellite. This percentage was higher than the genome coverage estimated from FISH (above), possibly indicating that the BAC library is biased for centromeric DNA, that short stretches of *CenSol* are scattered throughout the *S. invicta* genome, or both.

We next used the *S. invicta* A1repV1 primer pair to clone *CenSol* elements from the *S. geminata* genome. Electrophoresis of PCR products revealed a ladder-like pattern, consistent with *CenSol* being arranged tandemly in the *S. geminata* genome (Fig. 5b). The consensus sequence for each fire ant was generated using 45 units from the *S. invicta* BAC clones (above) and 52 units from the *S. geminata* clones. Sequence analysis showed that most of the repeats from both species carried the recognition site for the restriction enzymes EcoRV (GATATC) and BsaAI ((C/T)ACGT(A/G)) with a modal length of 109 bp (range: 108 bp to 122 bp; Additional file 4: Figure S1A-C).

We performed a multiple sequence alignment using the same sets of cloned sequences from *S. invicta* and *S. geminata*. We used the maximum likelihood method implemented in MEGA (1000 bootstrap replicates) to construct a gene tree for these *CenSol* sequences. These analyses revealed that the gene sequences from each species did not cluster into species-specific groups (Additional file 4: Figure S1D). This result indicates only minor divergence between the two *CenSol* sequence sets of the two ant species.

**Fig. 4:**
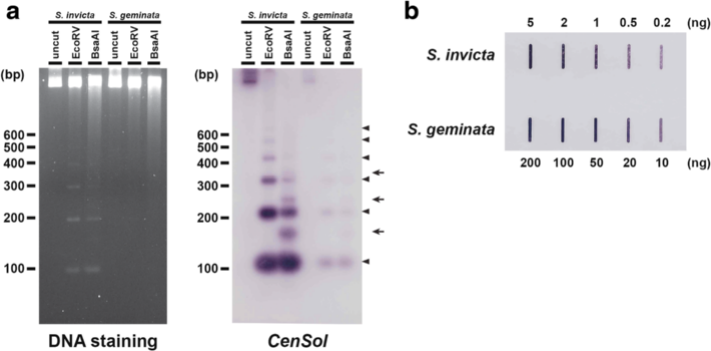
The abundance of the *CenSol* repeats in fire ant genomes. **a** Detection of the *CenSol* repeat by restriction digestion of *S. invicta* and *S. geminata* genomic DNA. Equal amounts of uncut or restriction digested (EcoRV or BsaAI) genomic DNA were separated by agarose gel electrophoresis and then stained with SYBR Safe (left panel). Southern analysis of the DNA from the left panel transferred to a nylon membrane with the *CenSol* probe (right panel). Two types of ladder-like patterns (arrowheads, main repeat; arrows, ‘off’ ladder repeats due to a BsaAI polymorphism) are indicated. **b** Slot blot hybridization to estimate the relative amounts of the *CenSol* sequence between the *S. invicta* and *S. geminata* genomes. DNA amounts are labeled

We used BLASTN to query the consensus sequence against the *S. invicta* [19] and *S. geminata* draft genomes and found 46,990 and 13,221 matches in the respective genomes, with some polymorphism in both length and sequence, in total covering ~4.1 and ~1.2 Mb. These values likely considerably underestimate the total *CenSol* coverage because repeats generally are collapsed in genome assemblies derived from short sequencing reads. The modal length of the *CenSol* BLASTN matches was 109 bp for both ant species (Fig. 2b). Similar to the cloned products above, nucleotide substitutions, insertions, and deletions were present among different monomers with some creating polymorphisms in the presence of restriction endonuclease recognition sites (e.g., EcoRV and BsaAI). This length variation also explains *CenSol* multimerization and the additional ‘off’ ladder steps in the restriction digestions (below, Fig. 4a).

Considering only the 109 bp monomers, there were 13,013 and 5720 copies (10,469 and 5423 unique) in the *S. invicta* and *S. geminata* genomes, respectively (Additional files 5 and 6). Pairwise comparisons of these unique 109 bp monomers using BLASTN [30] revealed that the nucleotide identity between species ranged from 70.6 to 99.1 % (intra-species comparison: 70.6 to 99.1 % in *S. invicta*; 71.6 to 99.1 % in *S. geminata*). We used WebLogo to summarize the aligned sequences and found that the nucleotides at each position were generally identical between *S. invicta* and *S. geminata*, with a few sites having a different predominant nucleotide (Fig. 2c). However, the two species did not share any identical *CenSol* sequences. The most similar pair between the two species differs at one base; position 88 is a thymine (T) in *S. invicta* and an adenine (A) in *S. geminata*. These data indicate that the *CenSol* repeat sequences are polymorphic but also highly similar between these species. Taken together, the conserved 109 bp *CenSol* repetitive sequence is the basic satellite DNA unit in both *S. invicta* and *S. geminata* genomes.

### *CenSol* copy number variation in *S. invicta* and *S. geminata*

We performed genomic restriction fragment analysis followed by gel electrophoresis to determine if *CenSol* repeats are arranged tandemly in both fire ants. The EcoRV digestion pattern of *S. invicta* genomic DNA displayed a ladder-like pattern with ~100 bp intervals (Fig. 4a, left panel, lane 2). Similarly, the BsaAI digestion pattern also produced an ~100 bp ladder-like pattern, with additional bands in between the main ~100 bp fragments (Fig. 4a, left panel, lane 3), which was due to the presence of infrequent BsaAI recognition sites created by sequence polymorphisms in the *CenSol* repeats (Fig. 2c). In contrast to *S. invicta*, no apparent restriction fragments were visible by DNA staining of the *S. geminata* genomic DNA digestions even though the same amount of DNA was used (Fig. 4a, left panel, lanes 4–6).

Southern hybridization using *CenSol* DNA confirmed that these restriction fragments were *CenSol* elements in *S. invicta* (Fig. 4a, right panel, lanes 1–3). Notably, this also revealed a clear ladder pattern in *S. geminata* (Fig. 4a). The similar restriction pattern in the *CenSol* Southern hybridization experiment between *S. invicta* and *S. geminata* confirmed that this satellite is arranged tandemly on chromosomes. Also the differences in DNA staining and hybridization signals indicate that the *S. invicta* genome has more *CenSol* units compared with the *S. geminata* genome. Thus, both ant species have the same or similar *CenSol* satellite but at different copy numbers.

We performed a slot blot hybridization experiment to quantify the difference in the relative amounts of *CenSol* repeats between the *S. invicta* and *S. geminata* genomes. A comparison of the *CenSol* hybridization intensity on different dilutions of genomic DNA revealed that the *CenSol* copy number in the genome of *S. invicta* was 10–20 fold more than that in *S. geminata* (Fig. 4b). The weaker hybridization intensities in *S. geminata* are not likely due to divergence of the *CenSol* sequence because this probe (although it is an *S. invicta CenSol* copy) has a high average similarity to the ensemble of copies for both species (~87 %). Thus, the slot blot results in combination with the restricted *CenSol* hybridization patterns in the FISH experiment reveal that the *CenSol* satellite is present but is far less abundant in the *S. geminata* genome.

### *CenH3* copy number in fire ants

Legume species with two copies of the *CenH3* gene have larger centromeres than those with a single copy [9]. We searched for *CenH3* paralogs in the *S. invicta* and *S. geminata* genomes to determine if either has additional *CenH3* gene copies. We found only a single copy of the *CenH3* gene in both genomes, and the gene sequences of both are most similar to the predicted *CenH3* genes of other insect species (Additional file 4: Figure S3). The nearest similar sequence was the canonical histone H3, as predicted. Thus, there is no clear association between the *CenH3* gene copy number and centromere size in fire ants.

### *CenSol* copy number evolution in other *Solenopsis*

Centromeric satellites may undergo differential expansion or contraction in closely related species [31]. We examined five additional species to determine how *CenSol* copy number has evolved in this group of ants. Altogether, we examined: three socially polymorphic fire ants from South America *S. invicta*, *S. macdonaghi*, and *S. richteri* (these three species belong to a single clade, and their colonies are either monogynous with one queen or polygynous with many queens, and this difference is genetically regulated [32, 33]); the social parasite fire ant *S. daguerrei*; two North American fire ants *S. aurea* and *S. geminata*; and a more distantly related thief ant *S. indagatrix* (outgroup). *S. geminata* forms a monophyletic clade with *S. aurea*, and this clade is sister to the group with *S. daguerrei, S. invicta*, *S. macdonaghi*, and *S. richteri* [33, 34].

**Fig. 5:**
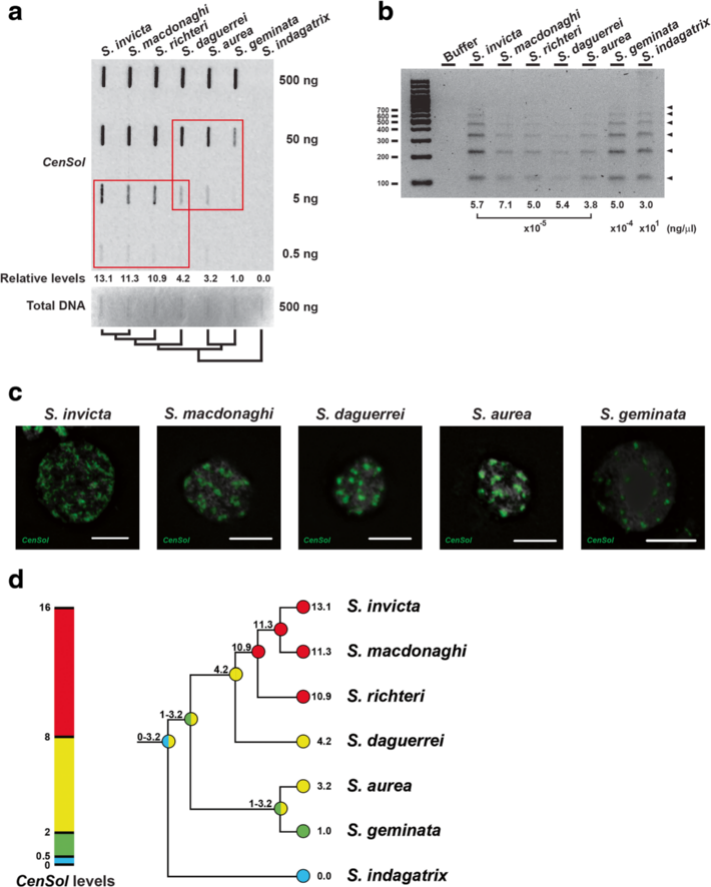
Evolution of *CenSol* copy expansion in *Solenopsis*. **a** Slot blot hybridization to estimate the relative amounts of the *CenSol* sequence among seven genomes. The *CenSol* signals were normalized with the staining intensities of total DNA (500 ng) by methylene blue. The *CenSol* signals at unsaturated dilutions for each species (red boxes, 50 ng and 5 ng, rows 2 and 3) were first calculated relative to the shared *S. daguerrei* genome and then rescaled relative to the *S. geminata* genome. DNA amounts are labeled. The evolutionary relationships of the seven species are shown below. **b** PCR assay for detecting *CenSol* satellite in seven *Solenopsis* species. Genomic DNA concentrations used for PCR are indicated below. **c** Localization of *CenSol* by FISH on diploid interphase cells in five *Solenopsis* species. **d** A model for the evolution of *CenSol* copy number in *Solenopsis*. The *CenSol* levels for the fire ant species (numbers at tips; values from (**a**) were used to infer ancestral state levels for *CenSol* (numbers above nodes) using the linear-change parsimony model. Continuous *CenSol* values were manually split into four bins (colors), which placed the seven studied species into four groups based on their relative *CenSol* levels

We estimated relative *CenSol* copy number differences using slot blot hybridization. We detected *CenSol* hybridization signals in six of the seven *Solenopsis* species (Fig. 5a). *S. invicta*, *S. macdonaghi*, and *S. richteri* contained comparable large numbers of *CenSol* repeats relative to each other, and 10.9 to 13.1 fold more than that of *S. geminata* (Fig. 5a, columns 1–3 and 6). The *CenSol* copies of *S. daguerrei* and *S. aurea* showed a moderate increase (4.2 and 3.2 fold) relative to *S. geminata* (Fig. 5a, columns 4–6). We did not detect *CenSol* in the outgroup species *S. indagatrix* using the slot blot assay (Fig. 5a). However, we could amplify the *CenSol* satellite by PCR using a high concentration of template DNA (~10^5^–10^6^ fold more). This suggests that a trace amount of *CenSol* satellite is present in the *S. indagatrix* genome (Fig. 5b). The PCR assay also revealed a ladder-like pattern of *CenSol* with ~100 bp interval in all seven species (Fig. 5b), which resembled the restriction digestion pattern of the *S. invicta* and *S. geminata* genomes (Fig. 4a). These data suggest that the *CenSol* repeats are distributed tandemly in all seven *Solenopsis* species.

We used FISH to compare the *CenSol* distribution for six of the seven species assayed by slot blot hybridization. We could not include *S. richteri* because we lacked tissue samples. Also, we decided to examine interphase cells for *S. macdonaghi*, *S. daguerrei*, *S. aurea,* and *S. indagatrix* because fresh ant samples of these species with dividing cells were not available. The *CenSol* signals were widely distributed on *S. invicta*, *S. macdonaghi*, *S. daguerrei*, and *S. aurea* chromatin, which parallel the expanded *CenSol* copies in their genomes (Fig. 5a). In contrast, *S. geminata* exhibited more localized signals of *CenSol* (Fig. 5c), which is consistent with the restricted *CenSol* signals on metaphase chromosomes (Figs. 2a, 3a, and Additional file 4: Figure S2A) and the weak signals in the hybridization experiments (Figs. 4 and 5a). Lastly, *CenSol* signals were undetectable by FISH for *S. indagatrix* (Additional file 4: Figure S4B), which is consistent with the absence of signal in the slot blot assay and the requirement of high template amounts for the PCR assay.

The long centromeres in *S. invicta*, *S. macdonaghi*, and *S. richteri* could be due to expansion from an originally shorter centromere; alternatively long fire ant centromeres contracted in the four other fire ant species. We conducted an ancestral state reconstruction analyses to infer which condition might have been more likely. Our analysis using the linear-change parsimony model indicates that the ancestral centromere state was short or moderate in length, supporting centromere expansion in the lineage leading to *S. invicta*, *S. macdonaghi*, and *S. richteri* (Fig. 5d). However, the direction of centromere evolution for *S. indagatrix*, *S. aurea*, and *S. geminata* was unresolved. We obtained a similar ancestral state pattern using squared-change parsimony (Additional file 4: Figure S5).

## Discussion

### Centromeres and centromeric satellite DNA in ants

Studies on ant chromosomes were led largely by Imai, Crozier and their co-workers starting in the mid-20th century [35]. As FISH was not available at that time, the vast majority of these cytological studies focused on the chromosome number and the karyotype [36, 37]. Despite knowledge of the karyotypes for >750 ant species [37], detailed examination of the centromere and identification of centromeric satellite sequences are lacking.

To the best of our knowledge, this is the first study combining bioinformatics and cytologicial examination of candidate centromere sequences in any ant. Previously, candidate centromeric repeats of four ant species were identified using computational methods to find the most dominant satellite in the genome [20]; however, no cytological evidence was provided to support centromeric localization of these satellites. We used a similar bioinformatics approach to identify the top ten satellites for two fire ant species, *S. invicta* and *S. geminata*. We then used FISH to demonstrate that the most common tandem repeat, *CenSol*, is localized to the centromeres on all chromosomes in both fire ants, whereas the second-most abundant satellite does not. Our results support that the *CenSol* satellite is a major component of fire ant centromeres, however definitive evidence will require CenH3 localization and chromatin immunoprecipitation studies (CenH3 antibodies are not yet available for fire ants).

### Unusually long centromeres in *S. invicta*

We demonstrated that the *S. invicta* centromeres are unusually long, spanning ~34 % of each chromosome. Such long centromeres have been found previously in only a few cases in plants and animals [4–10]. In contrast, the *S. geminata* chromosomes (except one) have the more typical, narrow centromeres commonly found in many animals and plants. We did not directly examine centromere length in *S. macdonaghi* and *S. richteri*, but they likely have long centromeres as well because both have high *CenSol* copy numbers and have *CenSol* interphase FISH patterns that were qualitatively similar to *S. invicta*. Correspondingly, *S. daguerrei* and *S. aurea*, which have ~4-fold greater *CenSol* copy number than *S. geminata* but less than *S. invicta*, *S. macdonaghi*, and *S. richteri*, likely have centromere lengths intermediate to those of *S. invicta* and *S. geminata*. We found very low but detectable levels of *CenSol* in the outgroup thief ant, *S. indagatrix*, suggesting that *CenSol* is not part of its centromere (i.e., is a minor repeat elsewhere) or is too divergent with only a few copies remaining conserved enough for detection (i.e., *CenSol-indagatrix* is different from *CenSol-invicta*).

Based on the current hypothesis of the phylogeny of the study species, our data suggest that there have been at least two bouts of *CenSol* expansion in the *Solenopsis* genus (Fig. 5d). Ancestral state reconstruction supports one centromere expansion event occurring in the common ancestor of four South American fire ants (*S. daguerrei, S. invicta*, *S. macdonaghi*, and *S. richteri*) [33, 34], and another later at the base of *S. invicta*, *S. macdonaghi*, and *S. richteri* (Fig. 5d). Centromere evolution in *S. geminata, S. aurea,* and *S. indagatrix* is unclear. Resolving whether expansion or contraction occurred in these three species (and throughout the genus) will require additional karyotypic studies of multiple *Solenopsis* species.

### A new centromere may be evolving in *S. geminata*

Our cytological studies showed that one *S. geminata* chromosome has an extended centromeric constriction (Fig. 1). Interestingly, *CenSol* hybridization was localized only to the edge of this constriction (Figs. 2a, 3a, and Additional file 4: Figure S2A), possibly indicating that a new centromeric satellite has invaded this *S. geminata* chromosome and may represent the early stages of a centromere revolution. Alternatively, it is possible that *CenSol* has not yet displaced the original centromeric satellite on this chromosome. The identity of this satellite remains to be determined, but our data demonstrate it is not the second most common repeat in the genome (see Fig. 2a).

### How did centromeres become so long in fire ants?

Centromeric satellite sequence turnover is well established and copy number differences of satellite repeats can be extreme both between species and among chromosomes within the same species [38–40]. However, why are all the centromeres so long in *S. invicta* (and in *S. macdonaghi* and *S. richteri*)?

One possibility is genetic drift whereby the copy number of *CenSol* on each chromosome increases or decreases by mutation (e.g., replication slippage) or recombination (e.g., unequal crossover) with copy number changes in either direction being equally likely and occurring independently for each chromosome. By chance all 16 of the *S. invicta* centromeres may have drifted to the longer sizes. A second possibility is that centromere length evolves neutrally but cell biological processes constrain all the centromeres to be of similar size, e.g., perhaps to avoid aneuploidy during cell division. While this may explain how all centromeres are uniformly long, it cannot readily explain the initial transition from the ancestral short to current long centromeres.

A third explanation, which we favor, is that selection for longer centromeres has occurred (and possibly is still ongoing), at least in some fire ants. We suggest centromere drive is likely the selective force underlying evolution of long centromeres in fire ants. Because only one of the four meiotic products in females is inherited by the egg, centromeres that violate the normally ostensibly fair process of meiosis have a selective advantage. The large sizes of all the primary constrictions in *S. invicta* chromosomes are consistent with a model of inter-homolog chromosomal competition [12]. Differences in centromere DNA length between the chromosome homologs may result in an uneven distribution of kinetochore proteins (e.g., CenH3), such that longer centromeres have more microtubule binding sites, and thus, preferential transmission into the oocyte. Because this is a general mechanism for all chromosomes, centromere drive could select for longer centromeres on every chromosome [12]. Thus, *S. invicta* and its relatives may have experienced runaway expansion of their centromeres.

The long *S. invicta* centromeres resemble the recently characterized metapolycentric centromeres in legumes and the compound centromeres in muntjacs and wallabies [5, 7–10]. These monocentric chromosomes have clear multiple centromeric protein docking domains, exhibiting either dotted or continuous patterns along the entire length of the constrictions. Metapolycentric chromosomes in legumes are associated with the maintenance of a duplicated copy of *CenH3* gene in the genome [9]. We found no evidence of additional copies of *CenH3*, and thus fire ant centromere expansion occurred through a mechanism independent of *CenH3* duplication.

### Could centromere expansions be a common feature of Hymenoptera?

Studies in the ciliated protozoan *Tetrahymena*, which only has female meiosis, has led to the proposal that species without male meiosis have unsuppressed (or at least relaxed selection on suppressors for) centromere drive, thereby facilitating greater centromere complexity and turnover [41]. In ants (and all Hymenoptera), males are haploid, and therefore, competition between centromeres is absent during male meiosis. Thus, selection for suppression of centromere drive during male meiosis would also be expected to be relaxed in Hymenoptera. This leads to an intriguing speculation that centromere complexity, expansion, and turnover in hymenopteran species is greater relative to species with both male and female meiosis.

One unanswered question is why haven’t long centromeres been reported more frequently in Hymenoptera? We suggest a reason is that long centromeres in Hymenoptera simply may have gone undetected as a result of previous karyotyping methods. Although there have been many karyotype studies in Hymenoptera, the vast majority of these cytological studies used colchicine or cocemid [28, 42], a mitosis inhibitor. These inhibitors arrest cells at metaphase, making it easier to find cells to karyotype, but prolonged exposure leads to artificially over-condensed chromosomes. Resolution of centromere morphology on such small chromosomes is difficult. Indeed the original karyotypes for *S. invicta* are tiny and long centromeres cannot be seen [27]. Additionally, given the frequent goal of categorizing centromeric locations (e.g., acrocentric, metacentric), there may have been inadvertent biases to focus on chromosome photos with well defined ‘X’ configurations. In this study we did not use a cell cycle inhibitor, a choice that likely considerably aided our ability to detect long chromosomes. We suggest that with better microscopy resolution and examination of less compacted chromosomes, more ants, other Hymenoptera, and other haplo-diploid organisms with long centromeres likely will be found.

## Conclusions

We describe a case of evolution of long centromeres in the fire ant *Solenopsis invicta*; centromere lengths are on average one-third of each chromosome. Several other species also have long centromeres while one species has the typical shorter centromeres. Expansion of this centromeric repeat likely occurred multiple times in fire ants. We also identified and characterized the major centromeric DNA repeat. Our results are consistent with a model of simple runaway centromere expansion due to centromere drive. We suggest that expanded centromeres may be more prevalent in ants, and other haplodiploid organisms, than previous considered.

## Methods

### Ant sampling

Three ant species were sampled in Taiwan: *S. invicta—*Taoyuan, *S. geminata—*Taichung and Tainan, and *S. indagatrix* – Nantou. The remaining species were sampled from: *S. richteri* —Pergamino, Argentina; *S. macdonaghi*—Antonio Joao, Brazil; *S. daguerrei*—Dourados, Brazil; and *S. aurea* —Indio, California. The *S. aurea* and three South American samples were stored in 95 % ethanol prior to analysis.

### Bacterial artificial chromosome (BAC) manipulations

In screening BAC clones for a previous study [18], clones A1, A5, and A8 (plate 73); B18 (plate 21); F22 (plate 68); and J6 (plate 7) of the SW_Ba BAC library (Clemson University Genomics Institute, Georgia, USA) were found to contain tandemly-repeated copies of the 109 bp *CenSol* sequence. To obtain DNA for end sequencing, these BAC clones were cultured in 12 or 48 mL LB medium containing 12.5 μg/ml chloramphenicol at 37 °C for 16 h and then purified using the Qiagen® Plasmid Mini Kit. Purified BACs were end sequenced with primers T7P and SP6. High-quality DNA sequences from the ends of these six clones (12 reads total) were chosen for *CenSol* sequence comparisons (see below). Centromeric monomer sequences are in Additional file 4: Figure S1A and Additional file 7.

To screen for the presence of the *CenSol* repeat sequences in other clones from the SW_Ba BAC library, a PCR assay was developed to amplify the repeat monomer (and its tandem multimers). Two primers, A1repV1F (5′-CGTGTTTTACGTTAAAA-3′) and A1repV1R (5′-TGAGATATCGCATAGATA-3′), were designed for the highly conserved region of the end sequences obtained from BACs A1, A5, and A8 (above). In total we screened an additional 960 clones (plates 96, 145, and half of plate 146) from the BAC library. We used a 384-well pin replicator to transfer approximately 1 μl of the thawed cultures from each clone to start 200 μl LB medium cultures containing 12.5 μg/ml chloramphenicol in 384-well deep well plates. BAC cultures were grown overnight at 37 °C and then 1 μl of the liquid culture was used for PCR amplifications of the repeat sequence. The PCR reactions were performed in 25 μl volumes containing 1X PCR buffer, 0.4 mM dNTPs, 0.2 μM A1repV1F and A1repV1R primers, and 1 U Super-Therm Gold Hot-start Taq DNA polymerase (JMR Holdings, Taiwan). PCR amplifications were performed with the following profile: initial 10 min denaturation at 95 °C; followed by 40 cycles of 30 s denaturation at 95 °C, 30 s annealing at 52 °C, and 30 s extension at 72 °C; and a final 7 min extension at 72 °C. PCR products were run on 1.5 % agarose gels containing SYBR® Safe DNA gel stain (Life Technologies) and visualized with the Quantum ST4-1000 gel imaging system (Vilber Lourmat).

### Bioinformatics analysis for tandem repeat identification and repeat clustering

We modified a previously developed bioinformatics pipeline [20] to identify potential centromeric sequences in the draft genome assemblies of *S. invicta* [19] and *S. geminata* (unpublished data). We searched for tandem repeats (satellites) with at least two copies within input scaffolds using Tandem Repeat Finder (TRF) [29] with parameters (Match = 1, Mismatch = 1, Indel = 2, Probability of match = 80, Probability of indel = 5, Min score = 200, Max period = 750) as described in [20]. We retained monomers greater than 50 bp and conducted an all-versus-all BLAST (megaBLAST) [43] against a database of dimer versions of monomers. We used SiLiX software [44] to group monomers (BLAST E-value < 1e-5 and ≥ 75 % sequence identity) into clusters. Custom Perl scripts were used to determine the total number of counts, the modal length, the number of counts for the repeat with modal length, and the GC content of each satellite for each species. To compare the top ten satellites between species, we conducted all-versus-all BLAST comparisons.

### Plasmid cloning and DNA manipulations

To obtain single *CenSol* repeats from *S. invicta,* we cloned random 109 bp fragments from an EcoRV digested BAC clone (A8 of plate 73) into plasmids. The *CenSol-F* (5′-ATCTCACGTGTTTTACG-3′) and *CenSol-R* (5′-ATCGCATAGATAGCGATTC-3′) primer pair and the p*CenSol-inv_4* plasmid template (Sinv_44 sequence in Additional file 4: Figure S1A and Additional file 7) were used for the *CenSol* probe amplification using the PCR DIG Probe Synthesis Kit (Roche). For *S. geminata*, we generated five *CenSol* satellite plasmids (p*CenSol-gem1-5*) by first using the A1repV1 primer pair to PCR amplify *CenSol* from genomic DNA and then cloning the PCR products greater than 2 Kb.

A single copy of *Solmin* (*Solenopsis* minor, the second most abundant satellite repeat) from *S. invicta* was cloned from a BspHI digested BAC clone (C16 of plate 73) into a plasmid, *pSolmin*. The primers *Solmin_F* (5′-TGATGGATCGAATCGCTA-3′) and *Solmin_R* (5′-TGAAAAAAGTTAAAACTC-3′) and the plasmid template were used for probe synthesis as above.

For Southern analysis, 200 ng of genomic DNA, extracted from single adult males of *S. invicta* and *S. geminata*, were digested with the EcoRV or BsaAI restriction enzyme (10 units) at 37 °C for 3 h. Uncut and digested DNA was separated by agarose gel electrophoresis and stained in-gel with SYBR® Safe (Life Technologies). The DNA in the agarose gel was transferred to a positively-charged nylon membrane (Biodyne B, Pall) by capillary transfer for Southern analysis [45]. Hybridizations were performed using DIG-labeled *CenSol* DNA. Alkaline phosphatase conjugated anti-DIG antibody combined with NBT/NCIP or CSPD substrate (Roche) was then used to detect the hybridization signals.

For the *CenSol* copy number comparisons between *S. invicta* and *S. geminata*, a slot blot with serially diluted genomic DNA from single adult males was used. For the comparison among the seven *Solenopsis* species, female pools (*S. invicta*, *S. geminata*, *S. macdonaghi*, *S. aurea*, *S. daguerrei*, and *S. indagatrix*) or a diploid male pool (*S. richteri*) was used. For both *S. invicta* and *S. geminata*, the samples for the two slot blots (Figs. 4b and 5a) were from different colonies, and those for the Southern analyses (Fig. 4a) were from a third colony*.* Hybridization and detection were performed as above for the Southern analyses. Methylene blue (0.02 % in 0.5 M sodium acetate, pH 5.2) staining was used to detect and control for the amount of loaded DNA.

To calculate the relative copies of *CenSol* in the seven genomes, we divided the *CenSol* hybridization signals by the staining intensities of total DNA. Due to the detection limit of methylene blue staining, we used the staining intensities at 500 ng for the seven genomes as the loading control for all respective dilutions. We calculated the relative amount of *CenSol* by averaging two unsaturated dilutions (Fig. 5a, red boxes). We used *S. geminata* as a reference to recalibrate their relative amounts. Signals on blots were digitalized by scanning (NBT/NCIP) or using the UVP Imaging system (CSPD and methylene blue staining) and quantified using the ImageQuant software.

### Fluorescence *in situ* hybridization (FISH) and image analysis

For the BAC-FISH analysis, we followed the instructions for probe preparation in the FISH Tag™ DNA Multicolor Kit (F32951, Invitrogen) with details indicated as follows. BAC clone A8 from plate 73 was used for the DNA template. Probe was generated by a nick translation reaction with amino-allyl modified dUTP (aa-dUTP) and labeled with the Alexa Fluor 488 fluorescent dye. For the *CenSol* and *Solmin* probes, the PCR products were amplified with a deoxynucleotide mixture containing aa-dUTP (aa-dUTP:dTTP = 6:1) and subsequently labeled with Alexa Fluor 488 and Alexa Fluor 594, respectively. Metaphase cells of *S. invicta* and *S. geminata* were collected from gonadal tissues of sexual brood at the 4^th^ instar larval stage; this stage is easily identifiable based on large size. Due to the absence of sexual brood in our *S. indagatrix* colony, metaphase cells were obtained from imaginal discs of worker brood at the 4^th^ instar larval stage. In addition we counted the chromosome number for ploidy. Chromosome spreads and FISH manipulations were as previously described [18]. Diploid interphase cells of *S. invicta* and *S. geminata* were from gonadal tissues of L4 larvae, whereas cells of *S. macdonaghi*, *S. daguerrei*, and *S. aurea* were from adult brain tissue. Chromosomes were counter-stained with DAPI fluorescent dye. Photos were captured using the DeltaVision imaging system and processed by deconvolution. Black and white images were false-colored (Alexa Fluor 488: green; Alexa Fluor 594: blue) and separate images were merged using Photoshop software.

The length of the primary constrictions and the coverage rate of *CenSol* signals on chromosomes of *S. invicta* and *S. geminata* were measured as previously described [9] for four images (cells) of a single haploid male by ImageJ software [46]. The DNA amount of the primary constrictions was calculated based on the proportion of DAPI fluorescence intensity within the primary constriction compared to that of the whole chromosome using an estimated haploid genome size of 484 Mb [19]. The centromeric positions were defined based on the arm ratio of chromosome termini to the edge of *CenSol* signal [47].

### Evolutionary analyses

Multiple sequence alignment (MSA) by ClustalW [48] was performed using 45 and 52 *CenSol* units with good Sanger sequencing traces from six *S. invicta* BAC clones and five *S. geminata* clones (*pCenSol-gem1-5)*. Gene trees were constructed using the Maximum Likelihood model and bootstrapping (1000 times) with the MEGA program [49]. The species-specific *CenSol* consensus clone sequences for *S. invicta* and *S. geminata* were generated from the MSA using VectorNTI software (Life Technology) and used as the input for BLAST queries [30] against the NCBI nucleotide nr database [50], Repbase [51], all candidate centromeric repeats [20, 52, 53], and the draft genomes of *S. invicta* and *S. geminata*. The 10,469 and 5423 unique sequences having exactly 109 bp from the BLAST results of the *S. invicta* and *S. geminata* genomes were used to construct sequence logos with WebLogo software [54].

The phylogenetic relationship of six *Solenopsis* species (*S. invicta*, *S. geminata*, *S. richteri, S. aurea*, *S. macdonaghi*, and *S. daguerrei)* was previously determined based on molecular evidence [33, 34]. We placed *S. indagatrix* as an outgroup to these six based on its status as a thief ant, morphology, and karyotype differences: *S. indagatrix* (1n = 11, Additional file 4: Figure S4A); *S. aurea*, *S. geminata*, *S. invicta*, and *S. richteri*, (1n = 16) [27, 28]. We used the quantification values of *CenSol* from the slot blot hybridization (Fig. 5a) as the input to reconstruct the ancestral state at all nodes of the tree in Mesquite 3.04 [55]. The state of *CenSol* values was coded as a continuous character. Ancestral states were calculated using both linear-change and squared-change parsimony in Mesquite 3.04 [55].

## Additional files

Additional file 1: Tandem repeat monomers identified from the *S. invicta* genome﻿. (TXT 2835 kb) Additional file 2: Tandem repeat monomers identified from the *S. geminata* genome. (TXT 1513 kb) Additional file 3: Table S1. Identification of the top 10 satellite families in the *S. invicta* and *S. geminata* genomes. (PDF 64 kb) Additional file 4: Figures S1-5. (PDF 1432 kb) Additional file 5: The unique set of 109 bp *CenSol* repeat sequences in the *S. invicta* genome. (TXT 1226 kb) Additional file 6: The unique set of 109 bp *CenSol* repeat sequences in the *S. geminata* genome. (TXT 634 kb) Additional file 7:
*CenSol* repeat sequences cloned from the genomes of *S. invicta* and *S. geminata*. (TXT 11 kb)

## Abbreviations

CenH3: Centromere-specific histone H3 variant
FISH: Fluorescence *in situ* hybridization
TRF: Tandem Repeat Finder
MSA: Multiple sequence alignment
BAC: Bacterial artificial chromosome
