# Supplementary material for: Evolution of long centromeres in fire ants
Source: BMC Evol Biol. 2016 Sep 15;16:189. doi: 10.1186/s12862-016-0760-7 (PMC5024525; doi:10.1186/s12862-016-0760-7)
Supplement: Additional file 3: Table S1. — Identification of the top 10 satellite families in the S. invicta and S. geminata genomes. (PDF 64 kb) [file 12862_2016_760_MOESM3_ESM.pdf]

Supporting table 1. Identification of the top 10 satellite families in the *S. invicta* and *S. geminata* genomes

S. invicta						
Rank	Family	Count	Modal_length (bp)	Modal_count	GC (%)	Similarity
1	FAM86	10824	109	7453	39.5	●
2	FAM361	822	139	271	31.6	■
3	FAM378	352	172	47	22.7	▲
4	FAM136	210	115	29	23.2	◆
5	FAM149	182	70	140	41.0	○
6	FAM233	162	52	36	43.0	□
7	FAM381	149	164	64	32.4	△
8	FAM1770	111	181	48	33.6	
9	FAM173	107	61	30	28.9	◇
10	FAM508	103	112	70	32.4	∗

S. geminata						
Rank	Family	Count	Modal_length (bp)	Modal_count	GC (%)	Similarity
1	FAM24	3005	109	2608	38.5	●
2	FAM52	688	138	224	32.2	■
3	FAM1	481	52	170	41.3	□
4	FAM104	318	164	228	32.9	△
5	FAM392	304	173	41	22.8	▲
6	FAM62	207	138	72	48.9	
7	FAM80	148	70	118	40.6	○
8	FAM91	141	62	105	34.6	◇
9	FAM40	141	115	18	23.7	◆
10	FAM101	101	112	80	32.9	∗
