# Supplementary material for: Evolution of long centromeres in fire ants
Source: BMC Evol Biol. 2016 Sep 15;16:189. doi: 10.1186/s12862-016-0760-7 (PMC5024525; doi:10.1186/s12862-016-0760-7)

Supplementary figure 1. The multiple sequence alignments (ClustalW) of *CenSol* for *S. invicta* (A) and *S. geminata* (B) was created using 45 and 52 units of *CenSol*, respectively. *CenSol* is defined as the DNA unit bounded by the A1repV1 primers with allowance for length or sequence variation. (C) Sequence alignment (ClustalW) of the *CenSol* consensus sequences (A and B). The locations of the A1repV1 primers are shown. (D) Gene tree of the *CenSol* repeats from the *S. invicta* and *S. geminata* genomes. The bootstrap values of each branch are shown accordingly. Sequences from *S. invicta* (blue circle); *S. geminata* (orange circle). Arrows in A and D indicate the *CenSol* sequence used in the Southern, slot blot, and FISH analyses.

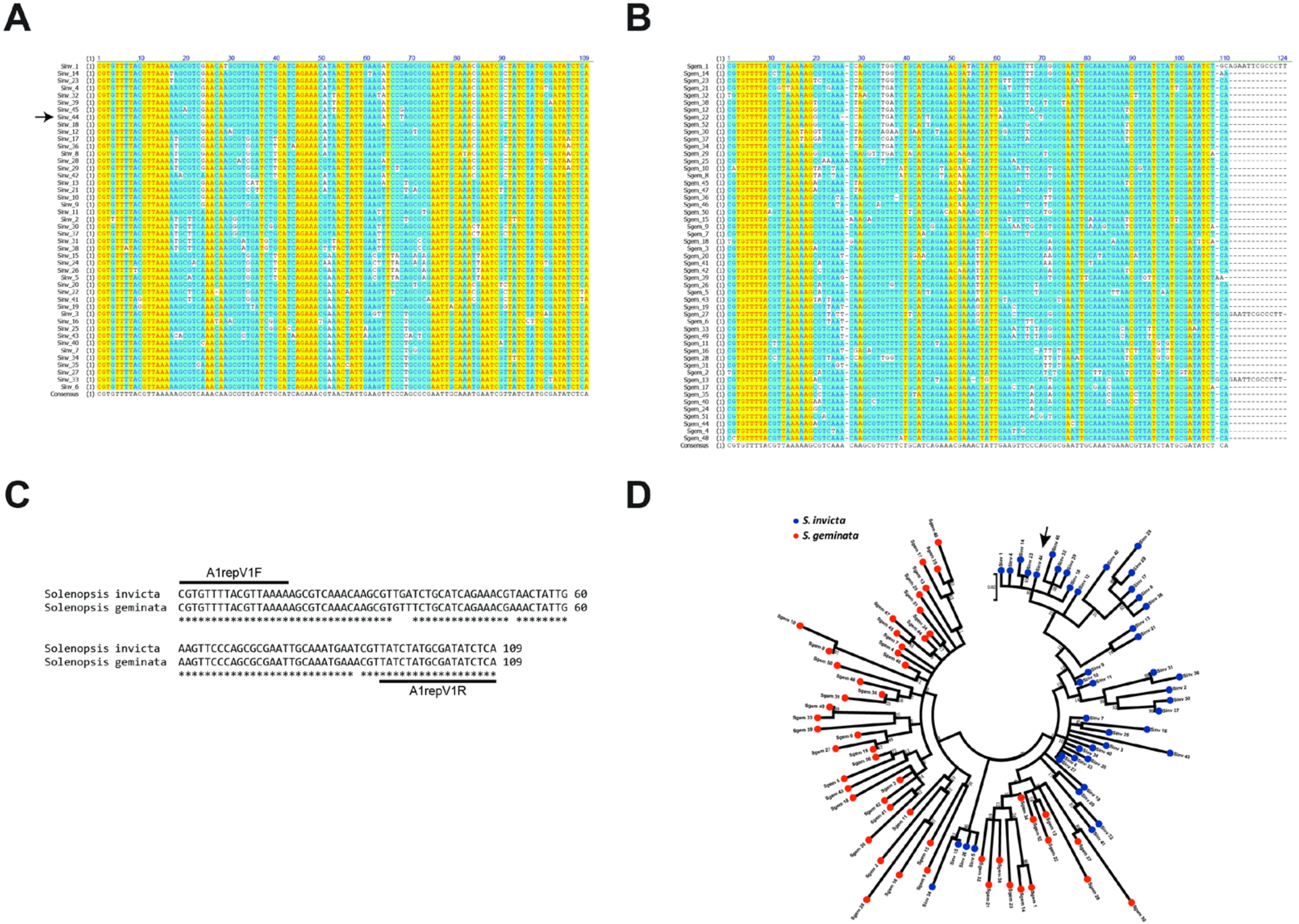
A
B
C
D
A1repV1F
Solenopsis invicta CGTGTTTTACGTTAAAAAGCGTCAAACAAGCGTTGATCTGCATCAGAAACGTAACTATTG 60
Solenopsis geminata CGTGTTTTACGTTAAAAAGCGTCAAACAAGCGTGTTTCTGCATCAGAAACGAAACTATTG 60
Solenopsis invicta AAGTTCCCAGCGCGAATTGCAAATGAATCGTTATCTATGCGATATCTCA 109
Solenopsis geminata AAGTTCCCAGCGCGAATTGCAAATGAAACGTTATCTATGCGATATCTCA 109
A1repV1R
S. invicta
S. geminata

Supplementary figure 2. Centromeric localization of the *CenSol* satellite repeat in *S. invicta* and *S. geminata*. (A) FISH analysis using a *CenSol* monomer. Two elongated primary constrictions (dimension lines) of a pair of chromosomes in *S. geminata* are indicated. (B) BAC probe on the *S. invicta* genome. (A and B) Metaphase chromosomes are from haploid males (1n=16) or diploid females (2n=32). Probes (green); chromosomes counterstained with DAPI (gray). Scale bars, 5 μm.

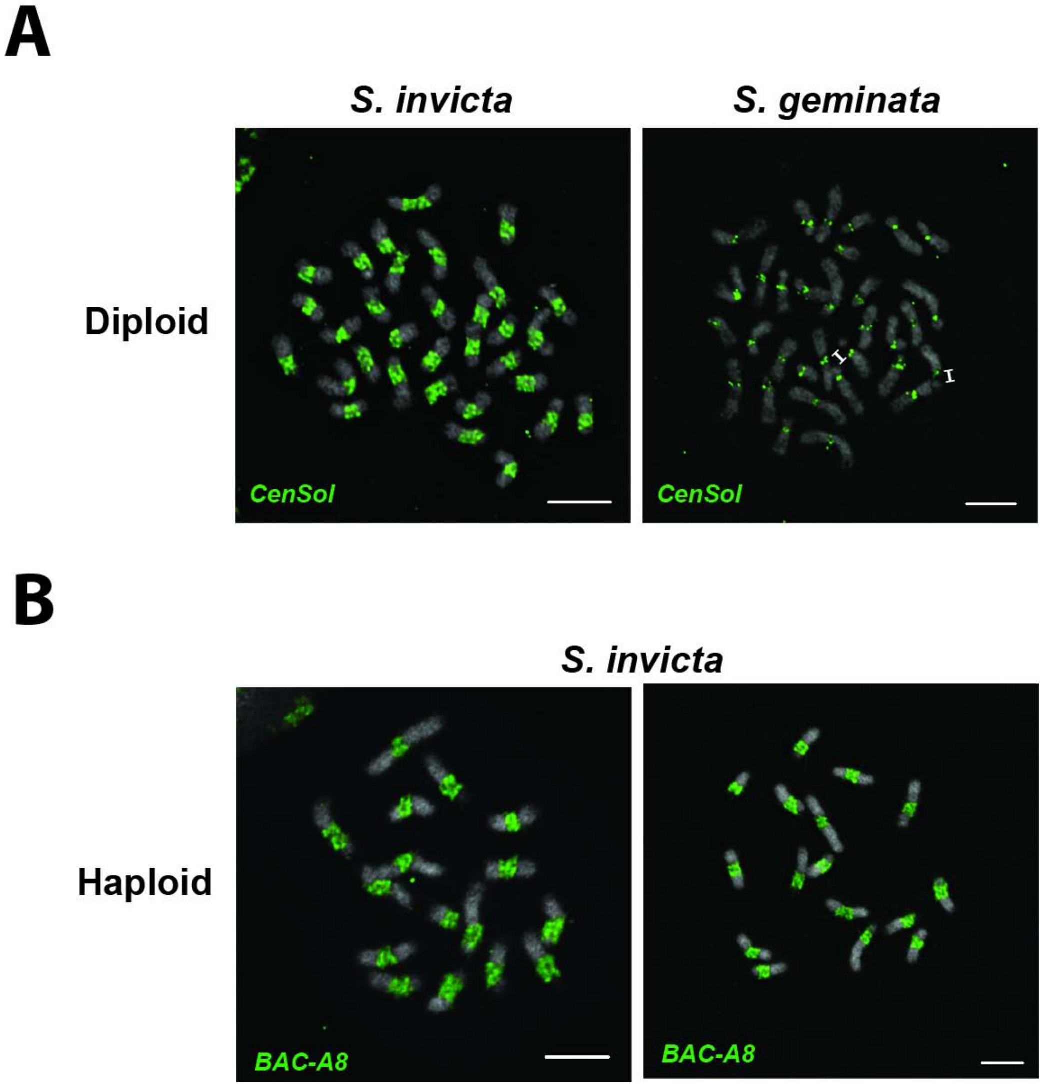

Supplementary figure 3. Phylogenetic analysis of the CenH3 proteins by using Maximum Likelihood Method. Numbers are with bootstrap values (%). The analysis was conducted with 15 insect species. Ants: *Atta cephalotes* (Acep), *Acromyrmex echinatior* (Aech), *Camponotus floridanus* (Cflo), *Cerapachys biroi* (Cbir), *Harpegnathos saltator* (Hsal), *Linepithema humile* (Lhum), *Monomorium pharaonis* (Mph), *Solenopsis geminata* (Sgem), *Solenopsis invicta* (Sinv), *Vollenhovia emeryi* (Veme), and *Wasmannia auropunctata* (Waur); wasps: *Microplitis demolitor* (Mdem), and *Nasonia vitripennis* (Nvit); sawfly: *Orussus abietinus* (Oabi); fruitfly: *Drosophila melanogaster* (Dmel).

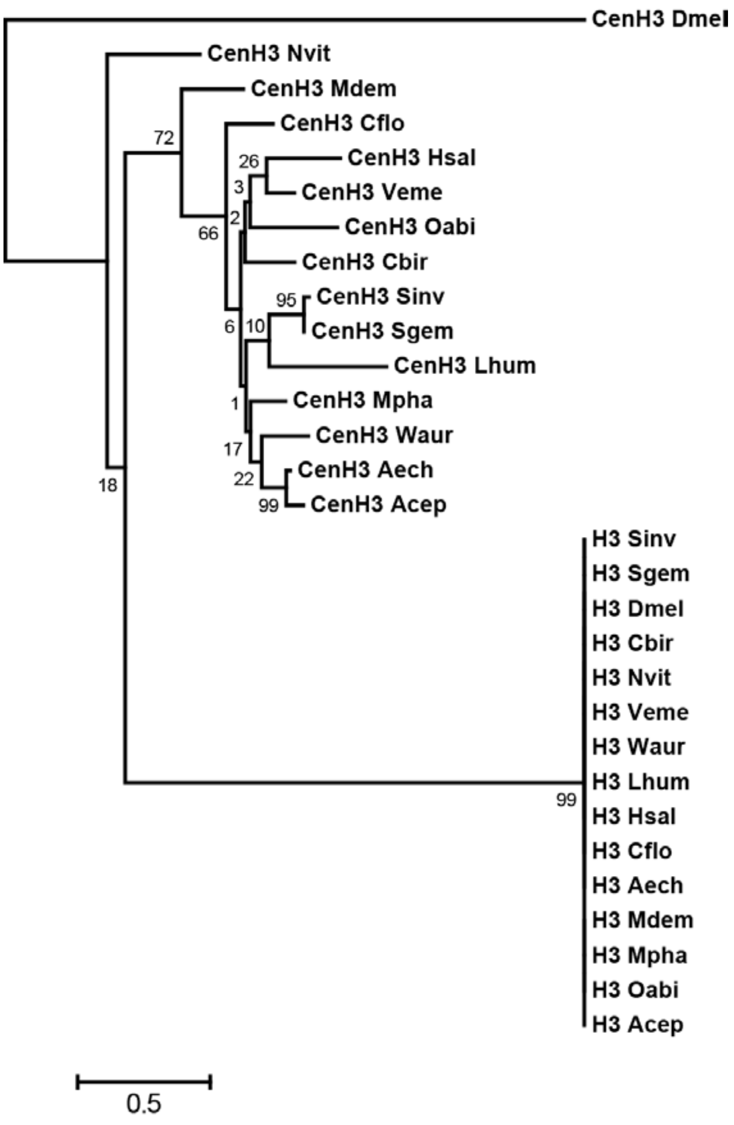

Supplementary figure 4. *S. indagatrix* karyotype and FISH analysis. (A) DAPI staining of *S. indagatrix* metaphase chromosomes from worker imaginal discs. (B) FISH analysis with the *CenSol* probe (green); chromosomes counterstained with DAPI (gray). Nucleus is marked by the dashed circle. No clear *CenSol* signal is present in the genome in comparison to the background noise.

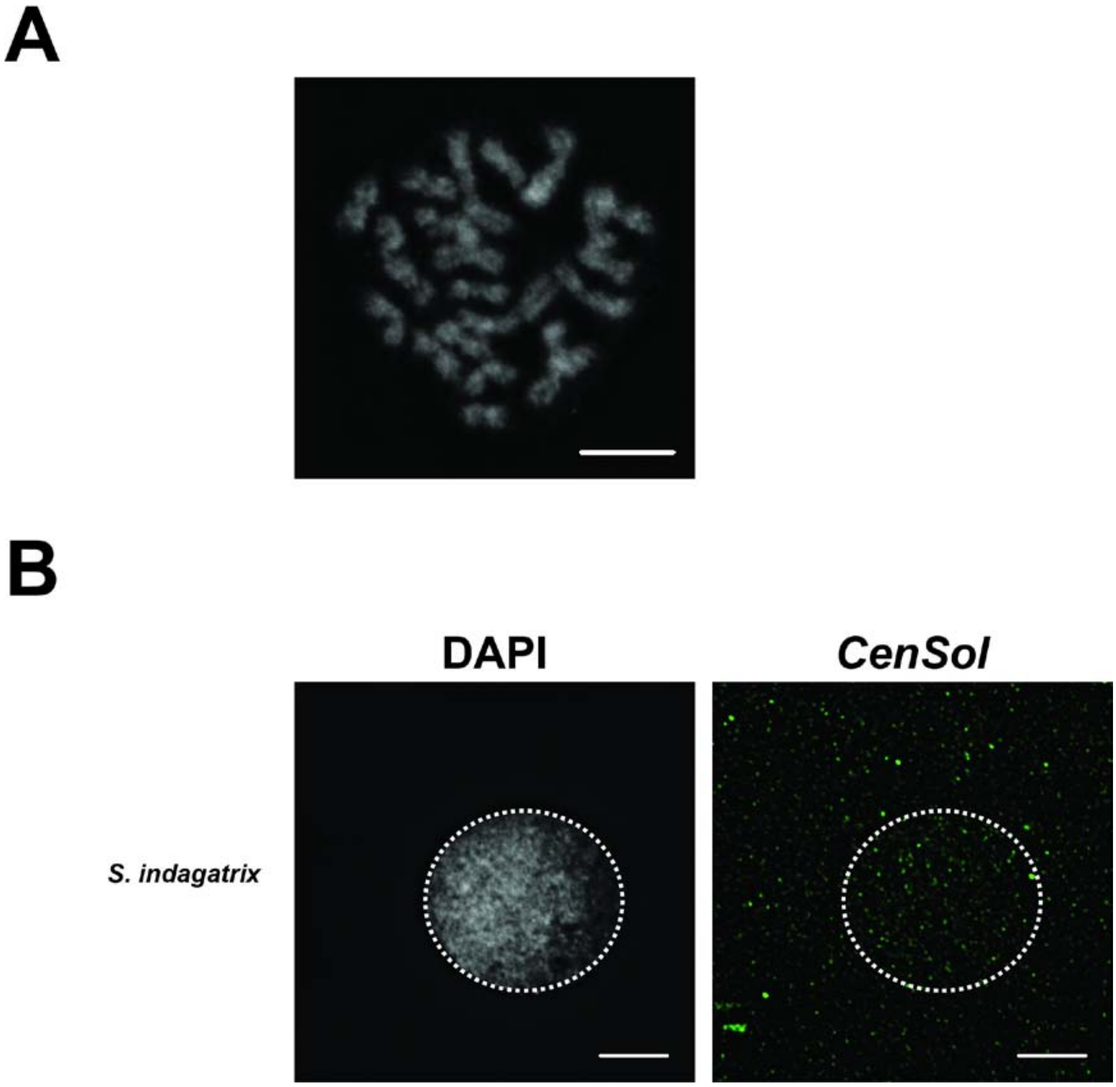

Supplementary figure 5. Ancestral state reconstruction of *CenSol* copy number in *Solenopsis* using the squared-change parsimony model.

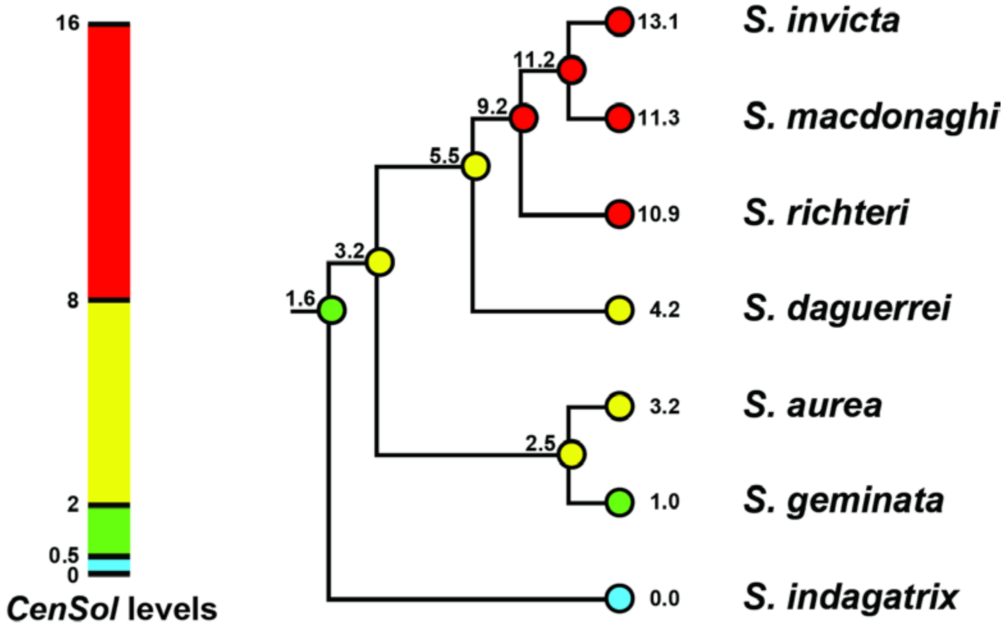

Supplement: Additional file 4: — Figures S1-5. (PDF 1432 kb) [file 12862_2016_760_MOESM4_ESM.pdf]
